# The Physiological and Agronomic Responses to Nitrogen Dosage in Different Sugarcane Varieties

**DOI:** 10.3389/fpls.2019.00406

**Published:** 2019-04-05

**Authors:** Yingying Yang, Shiwu Gao, Yong Jiang, Zhaoli Lin, Jun Luo, Mingjie Li, Jinlong Guo, Yachun Su, Liping Xu, Youxiong Que

**Affiliations:** ^1^Key Laboratory of Sugarcane Biology and Genetic Breeding, Ministry of Agriculture, Fujian Agriculture and Forestry University, Fuzhou, China; ^2^College of Crop Science, Fujian Agriculture and Forestry University, Fuzhou, China; ^3^College of Computer and Information Sciences, Fujian Agriculture and Forestry University, Fuzhou, China

**Keywords:** sugarcane, variety, physiological, agronomic, indicators, nitrogen use efficiency, screening, nitrogen fertilizer application

## Abstract

Nitrogen (N) is very important for sugarcane yield improvement, but the excessive application of N fertilizer brings about N pollution and a cost increase. Through distinguishing the difference of nitrogen use efficiency (NUE), we can reasonably apply N fertilizer according to the NUE characteristics of sugarcane varieties, and thus reduce N loss and maintain high yield. The present study showed the pot experiment results of identifying NUE types of nine main sugarcane varieties in the main sugarcane producing areas of China under controlled conditions, and identified the key physiological and agronomic indictors which can help to determine the NUE types of sugarcane. The test clones were exposed to varying levels of N fertilizer and 15 parameters that are likely to impact NUE were measured. The key results are (1) Sugarcane variety ROC22 has the high plant dry weight (PDW) and NUE among nine varieties under different N rates, it can take advantages under low N supply (225 kg/hm^2^ urea), and less N fertilizer can be applied properly in production. (2) Varieties of GT32 was good performing genotype for PDW and NUE under low N supply (225 kg/hm^2^ urea), GT42 was more suitable for moderate N environment (450 kg/hm^2^ urea), while YT94-128 was at middle N and high N supply (450–675 kg/hm^2^ urea). (3) Late stage of shoot elongation is suitable for differentiating sugarcane clones for NUE. (4) Leaf glutamine synthetase activity is the most reliable predictor of NUE in sugarcane. The result of pot experiment is sufficient to differentiate clonal variation for NUE in sugarcane as it reflects field experimental results. This study can set up a basis for identification the NUE types of sugarcane varieties and the development of reasonable N fertilizer application.

## Introduction

Sugarcane is the most important sugar crop, accounting for approximately 80% of sugar production in the world (Islam et al., 2018; Sharma and Chandra, 2018) and more than 90% of that in China (Li et al., 2016). Nitrogen (N) is a key restrictive factor for continuous sugarcane output. A proper amount of N-fertilizer can remarkably increase tillering and thus results in an early population with high yield, which can increase output (Gopalasundaram et al., 2012). Insufficient or ill-timed supply of N-fertilizer applied to sugarcane would result in poor growth, such as narrow leaves, thin stems, and short internodes (Bell et al., 2014), while excessive application of synthetic N-fertilizer leads to a cost increase, acidic soil, eutrophic water and non-point pollution (Ju et al., 2009; Chen et al., 2014) and finally decreases the sugar content (Muchow et al., 1996), especially at the sugar accumulation stage. This phenomenon is quite common in the two sugarcane producing countries, China and India, due to the continuous planting of sugarcane for many years, which resulted in a low output-input ratio, i.e., only 0.1 for China and 0.3 for India based on the International Fertilizer Industry Association Assessment of fertilizer use by sugar crops at the global level 2007/2008 (Robinson et al., 2011). In China, to help planters to obtain higher production, approximately 500–700 kg/hm^2^ of N fertilizer per single crop season has been applied, which is 2–3 times as much as that in Brazil and Australia (Yang et al., 2014; Li et al., 2015). N fertilizer recovery in sugarcane ranges from 20 to 40% (Meyer et al., 2007; Kingston et al., 2008; Franco et al., 2010), which is relatively low, and most of N is lost from soil (Chapman et al., 1994). The nitrogen use efficiency (NUE, crop production/fertilizer N supplied) of sugarcane genotypes varied greatly, and high NUE varieties are an important issue for sugarcane industry. One avenue helps to alleviate the excessive use of N is to determine the NUE of crops based on the various absorption and utilization characteristics of N-fertilizer and then the appropriate amount of N-fertilizer was applied (Hirel et al., 2001; Snyman et al., 2015). Therefore, the identification of NUE types of crops for rational N fertilizer application and how to identify a variety with high NUE under low N conditions are of great significance for reducing N pollution.

It is well-known that the N demand depended on the size of the crop and NUE (Ladha et al., 2005). The NUE level of the crop variety determinates the degree of N uptake and utilization. Matching N supply according to crop N demand is an important way to ensure crop yield and reduces the amount of N fertilizer applied (Bell et al., 2014). Many scholars had conducted researches on the NUE characteristics of crop varieties, and the varieties with higher NUE under different N condition were screened out. Cohan et al. (2018) assessed the potato cultivars effect on NUE components to provide farmers better advice on N fertilizer application rate in France. Cong et al. (2017) investigated the effects of different N fertilizer application on N use rate, grain yield and quality in different rice varieties, and found that OM052 was a variety with high N agronomic efficiency and yield. There were also similar reports in sugarcane. According to the kinetic indicators of N-uptake by different genotypes of sugarcane plantlets under different N-content solutions, high or low NUE sugarcane varieties were selected (Hajari et al., 2014). Robinson et al. demonstrated in their study that Q165A was good performing genotypes for biomass and iNUE (biomass produced per unit tissure N) at high N and low N solutions (Robinson et al., 2007). Both reports focused on sugarcane plantlet cultured in water. In order to get closer to production practice, we found out the varieties with higher NUE and plant dry weight (PDW) under low N conditions from main varieties planted in large areas at present China, and a certain/rational amount of N fertilizer was applied to achieve the purpose of maintaining crop yield and reducing N pollution.

There are large differences in NUE among various crops and varieties of the same crop, and the differences are closely related to the ability of N assimilation in plants (Ju et al., 2015; Wang et al., 2015; Chen et al., 2016). Normally, two forms of N, i.e., nitrate-N and ammonium-N, can be absorbed and assimilated by plants. After uptake, nitrate-N is catalyzed and transformed by nitrate reductase (NR) and nitrite reductase (NIR) to ammonium salt and then to amino acids. There are two main pathways for ammonium-N to be transformed into amino acids, one of which involves glutamate dehydrogenase (GDH), and the other involves glutamine synthetase (GS) and glutamate synthase (GOGAT) to produce glutamate (Lam et al., 1996). Several key catalytic enzymes of N assimilation are of great importance in the N use of plants. The differences in the key catalytic enzymes among various crops or varieties of the same crop affect N assimilation (Jawad et al., 2017). Key enzyme activities associated with N assimilation are important physiological indicators which have been used to explore NUE in many studies (Oliveira et al., 2013; Jawad et al., 2017). QTLs for NR, GS activity, grain N content and grain yield were co-localizations of field screening maize population in N assimilation enzymes activities, plant nitrate concentration and agronomic indictors, and locus on chromosome 5 was found to be a good candidate gene for explaining yield variations (Hirel et al., 2001). This illustrated that the reaction catalyzed by GS represented key element controlling NUE in maize. In barley, the increasing level of GS, GOGAT, and GDH activity in a plant with high NUE was higher than that with a low NUE (Jawad et al., 2017). Oliveira et al. (2013) found that GS activity of maize plantlets was a better indicator than NR activity for NUE. The activities of GS1 and GS2 could be a potential marker to predict and select NUE in wheat varieties (Zhang Z.Y. et al., 2017). Foulkes et al. (2009) suggested that leaf GS, leaf photosynthetic rate and stem N storage contributed to the variation in NUE among wheat varieties. GS activity could be an effective indictor for screening rice varieties with higher NUE (Rajesh et al., 2017). In addition, a strong relationship was obtained between leaf GS activity and total N content, but GDH activity had no significant correlation with the total N content in maize (Hirel et al., 2005).

In sugarcane, regarding the relationship between key enzyme activities of N metabolism and NUE, except for Robinson et al. (2007), who investigated the relationship between GS activity and NUE in sugarcane plantlets under water culture and found no correlation between GS activity in the leaf/root and NUE, a little of attention has been paid to the key enzyme activity of N assimilation to evaluate the NUE of sugarcane. Li selected sugarcane varieties plantlets with under low N treatment in sand culture, which suggested that above-ground biomass and the photosynthetic rate could be used as screening indicators for low-N tolerance at the early stage of sugarcane growth (Li, 2011).

In the present study, using pot and field experiments, we focused on the main sugarcane varieties with different NUEs in the present China, and investigated their physiological indicators and agronomic traits of aboveground and belowground at different synthetic N-fertilizer application rates. The objectives of this work were to (1) Distinguish the NUE and PDW difference of nine widely planted sugarcane cultivars under different dosage of N-fertilizer in China. (2) According to the NUE and PDW characteristics of varieties, suggestions on rational N fertilizer application rate were provided. (3) Determine the suitable growth stage for the investigation of parameters to predict NUE in sugarcane. (4) Evaluate indicators for predicting NUE of sugarcane.

## Materials and Methods

### Materials

The representative sugarcane varieties ROC16 (Yang et al., 2006), ROC22, GT32, FN41, GT42, YT94-128, LC05-136, NCo376 (Hajari et al., 2014) and Badila, with different NUEs, were selected. All of these varieties were provided by the Key Laboratory of Sugarcane Biology and Genetic Breeding, Ministry of Agriculture, Fujian Agriculture and Forestry University.

### Pot Experiment

The outdoor pot experiment was conducted from March 2016 to January 2017 at Fujian Agriculture and Forestry University, Fuzhou, China. The pots, which were 0.40 m in both diameter and depth, with eight 1.0-cm-diameter holes at the base, were filled with 10 kg of soil. The soils foundation fertility was assayed according to the method of Zhao et al. (2013). The pH was 5.0, the organic matter content was 7.09 g/kg, alkali-hydrolyzable N was 0.04 g/kg, effective phosphorus was 0.02 g/kg, rapidly available potassium was 0.10 g/kg, total N was 0.38 g/kg, total phosphorus was 0.28 g/kg and total potassium was 20.1 g/kg.

The experimental design was a double-factor completely randomized design. Factor A was sugarcane varieties (ROC16, ROC22, GT32, FN41, GT42, YT94-128, LC05-136, NCo376, and Badila). Factor B was N treatments (Zhao et al., 2014; Li et al., 2015): N1 was 225 kg/hm^2^ urea, N2 was 450 kg/hm^2^ urea, N3 was 675 kg/hm^2^ urea, and no N fertilizer (0 kg/hm^2^ urea) was established to calculate NUE of N1, N2 and N3 treatments, with urea as the N source. The experiment consisted of 36 treatments and 12 pots per treatment. Others fertilizers (besides N fertilizer) were applied similarly to each pot. Fertilizer: urea (total N ≥ 46.4%, China), calcium magnesium phosphate fertilizer (P_2_O_5_ ≥ 12.0%, China) at 925 kg/ha, potassium chloride (K_2_O ≥ 62.0%, Russia) at 335 kg/ha. According to the field plantlet number (90,000 plantlets/ha) and fertilizer requirement, the dose of urea of the four N treatments was 0.00 g for N0, 2.50 g for N1, 5.00 g for N2 and 7.50 g for N3 in each pot. A total of 30% of the N fertilizer was applied as basal fertilizer, and the remaining 70% was applied as a topdressing in late July 2016. The dose of calcium-magnesia phosphate fertilizer and potassium fertilizer was 10.28 and 3.72 g per pot, applied as basal fertilizer.

The relatively consistent sugarcane plantlets at three-leaf stage were transplanted manually on 20 April 2016, and one plantlet was planted in each pot. All planting pots were poured the same amount of water, and were well-watered during the experiment. The daily cultivation management of each pot was the same.

When we sampled the roots, we first filled the whole cultivation pot with water and soaked for 6 h, then carefully removed the whole plant along with the roots, and finally put it in a large tub and rinsed carefully until the root system was washed out. Three biological repeats were used for measurements at each indictor, and each plant is as a biological repeat.

### Field Trial Design

The field experiment was carried out from March 2017 to January 2018 in the experimental field of Fujian Agriculture and Forestry University, Fuzhou, China. The experimental design was a split plot and consisted of 36 treatments. The main plot was divided into four N treatments: N1 was 225 kg/hm^2^ urea, N2 was 450 kg/hm^2^ urea, N3 was 675 kg/hm^2^ urea, and no N fertilizer (0 kg/hm^2^ urea) was established to calculate NUE, with urea (total N ≥ 46.4%, China) as the N source. The subplots corresponded to nine sugarcane varieties, namely, ROC16, ROC22, GT32, FN41, GT42, YT94-128, LC05-136, NCo376, and Badila. Each treatment had four 8 m-long rows with a row spacing of 1.3 m, with three replications. The field plantlet number was 90,000 per hectare. Determination of soil basic fertility used the method of Zhao et al. (2013), the soil pH was 5.6, the organic matter content was 14.2 g/kg, alkali-hydrolyzable N was 0.06 g/kg, effective phosphorus was 0.04 g/kg, rapidly available potassium was 0.06 g/kg, total N was 0.80 g/kg, total phosphorus was 0.69 g/kg and total potassium was 24.8 g/kg. The fertilizer application amount and application method of N, calcium-magnesia phosphate and potassium were the same as in the pot experiment. Sugarcane was planted manually on 7 March 2017, and the cultivation management was similar to the field production of sugarcane.

### Measurements

The plant height (H), stalk diameter (D), plant fresh weight and physiological indexes were measured in early July (the early elongation stage, Stage 1) and early October (the late elongation stage, Stage 2) of 2016 in pot experiment, and physiological indicators include the activities of GS, GDH and GOGAT, soluble protein content (SPC), net photosynthetic rate (Pn), leaf chlorophyll relative content (single-photon avalanche, SPAD), Chlorophyll fluorescence.

The activity of GS, SPAD values and Plant fresh weight were measured at Stage 2 of 2017 in field trial. N contents were measured in Stage 1, Stage 2 and early January of second year (the technical maturation stage, Stage 3) in pot experiment, and Stage 3 in field trial.

The middle part of the first fully expanded leaf was taken as the measuring site for photosynthesis, fluorescence parameters, SPAD values, activities of GS, GDH, GOGAT, SPC and expression analysis of *GS* family genes by qRT-PCR. After measuring the photosynthesis and fluorescence parameters of leaf, the middle part of leaf was taken immediately, washed thoroughly with distilled water, and divided into two parts. One part of leaf measured the key enzymes activities in N metabolism. The other part of leaf was frozen immediately in liquid nitrogen for expression analysis of *GS* family genes by qRT-PCR.

#### Photosynthesis Indices

Net photosynthetic rate (Pn) was measured using an LI-6400XT portable photosynthesis system (Li-COR, Lincoln, NE, United States) between 8:00 and 11:00 a.m. under cloudless sky. When measuring leaf Pn, blue light of 1,500 μmol m^-2^ s^-1^ was provided. Relative humidity was adjusted to 70%. The leaf chamber CO_2_ concentration was set to 400 ± 5 μl L^-1^, and the leaf temperature was set to 29 ± 2°C.

#### SPAD Values

According to a previous experimental method (Dinh et al., 2017), SPAD values was estimated with a SPAD-502 Plus (Konica Minolta Sensing, Inc., Osaka, Japan). After photosynthesis measurements, SPAD values were recorded at the same position.

#### Chlorophyll Fluorescence

According to the experimental method of Shu et al. (2016), chlorophyll fluorescence was measured using an IMAGING-PAM fluorometer (Walz, Effeltrich, Germany) on individual leaves, previously used to measure SPAD values, following a dark adaptation period of 20 min. The Fv/Fm of chlorophyll fluorescence (the maximum quantum yield of PSII, which represents the ratio between variable and maximum fluorescences, Fv/Fm) was calculated using the fluorometer’s software. The measured location of Chlorophyll fluorescence was the same as that of photosynthesis indices and SPAD values.

#### Key Enzymes Activities of N Assimilation

The middle part of the first fully expanded leaf and fibrous root of each sampled plant was cut to determine key enzymes activities of N assimilation in the pot and field experiments. According to the method of Wang et al. (2014), GS activity was measured. One unit of GS activity (U) was defined as a 0.01 change in A_540_ per minute per ml reaction system. GOGAT and GDH activities were measured according to the experimental method of Groat and Vance (1981). One unit of GOGAT was expressed as the amount of enzyme that catalyzed the oxidation of 1 nmol NADH per min. GDH activity was determined by recording the reduction of NAD (deaminating GDH activity, NAD-GDH) or the oxidation of NADH (aminating GDH activity, NADH-GDH). One unit of GDH was calculated in units of nmol of NADH oxidized/NAD reduced per minute. SPC of the leaf and root was measured following the experimental method of Laukkanen and Sarjala (1997).

#### Expression Analysis of Five *GS* Family Genes in Sugarcane by qRT-PCR

Total RNA of each sample was extracted with TRIzol reagent (Invitrogen, Shanghai, China), and RNA quality was examined using a NanoDrop (Thermo Fisher Scientific, Inc.) and an Agilent 2100. Bioanalyzer (Agilent Technologies, Santa Clara, CA, United States). According to the research of Nogueira et al. (2005), *GS1.a*, *GS1.b*, *GS1.c*, *GS2*, and *GSI* were selected and their expressions were analyzed by qRT-PCR using SYBR Green staining. Quantitative specific primer of *GS1.a*, *GS1.b*, and *GS1.c* were reference to Nogueira et al. (2005), and primers of *GS2* and *GSI* were designed using NCBI primer designing tool^1^. The sequences of primers are shown in Supplementary Table S1. The glyceraldehyde-3-phosphate dehydrogenase gene (*GAPDH*, GenBank Accession No. CA254672) was used as the internal reference gene for qRT-PCR (Ling et al., 2014). Three biological replicates were performed for each treatment. 2^-ΔΔCt^ was used for the calculation of qRT-PCR data (Livak and Schmittgen, 2001).

#### Determination of N Content

Four individual parts of the plant, leaf, leaf sheaths, stem and root, were sampled and rinsed with pure water. All plant samples were dried in an oven for 3 h at 105°C and then for another 72 h at 80°C. PDW was calculated as the sum of the leaves, leaf sheaths, stem and root dry weight. The samples were finely ground with a mill (Shanghai Jingxin, Co., Ltd., China) and were sieved through a 0.25-mm mesh. According to the ratio of the dry weight of each individual part to PDW, 0.5-g samples were collected and then digested with H_2_SO_4_-H_2_O_2_ at 260–270°C for total N measurement. According to the Kjeldahl method reported by Cao et al. (2017), plant N content (PNC) was determined by a Kjeltec 8200 type automatic azotometer (Foss, Denmark). Plant N accumulation (PNA) was calculated as:

Plant N accumulation (PNA, g/plant) = PNC × PDW (Zhang C. et al., 2017)
NUE (gDWg^-1^N) = [shoot DW (g) of fertilized treatment-shoot DW (g) of 0 N treatment] ÷ amount (g) of N applied (Zhao et al., 2014)

Decline ratio of NUE (DRNUE) was calculated as:

DRNUE = (NUE of N2 treatment – NUE of fertilized treatment) ÷NUE of N2 treatment.

### Statistical Analysis

All statistics, including analysis of variance (ANOVA), significant differences, principal component analysis (PCA), correlation analysis and cluster analysis, were performed using the SPSS 19.0 system (SPSS, Inc., United States). The treatment means were separated using Duncan’s significant difference test, with *P* < 0.05 as the significance level.

## Results

### Effects on the Activity of Key Enzymes Involved in N Metabolism

#### The Early Elongation Stage (Stage 1)

**FIGURE 1:**
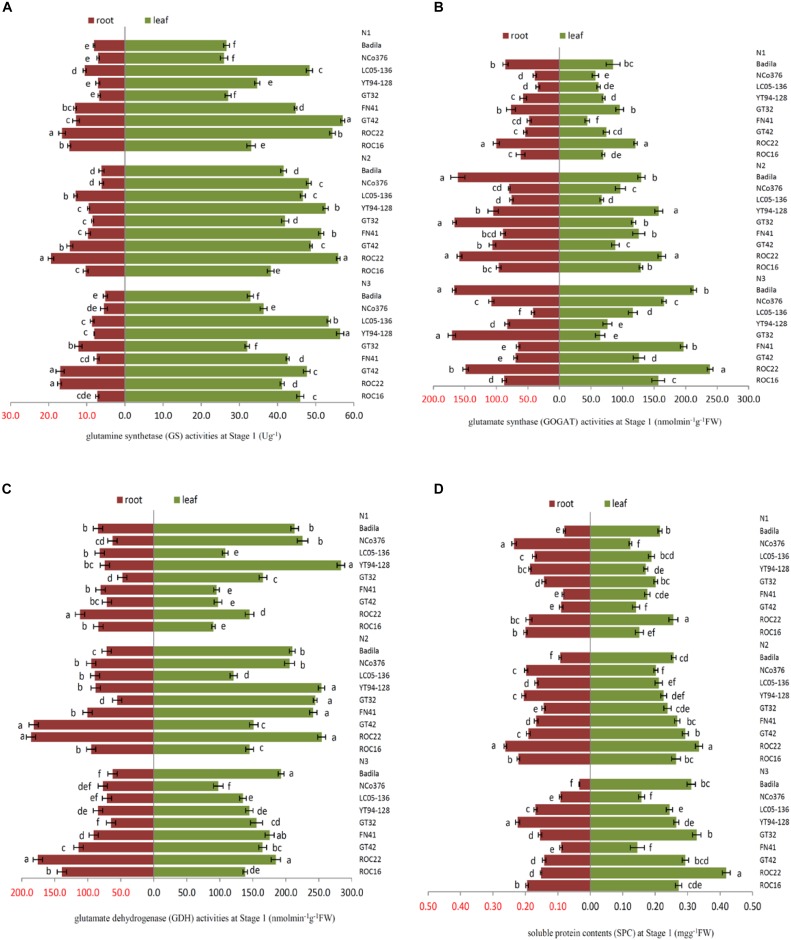
Effects of different N application rates on N assimilation enzyme activities at Stage 1 in 2016. **(A)** GS (glutamine synthetase activity), **(B)** GOGAT (glutamate synthase activity), **(C)** GDH (glutamate dehydrogenase activity), **(D)** SPC (content of soluble protein). N1 was 225 kg/hm^2^ urea, N2 was 450 kg/hm^2^ urea, N3 was 675 kg/hm^2^ urea. Stage 1, the early elongation stage. Different lowercase letters represent significant differences between the different varieties with respect to the same N application rates (*P* < 0.05).

It is indicated in Figure 1, the activities of GS, GOGAT, GDH, and SPC in leaves of the same sugarcane variety at different N application rates were significantly higher than those of roots at Stage 1 (*P* < 0.05). There were also obvious differences in the activities of different enzymes in response to N application. Compared with N2, N1 had the greatest effect on the GOGAT activity of roots and had minimal effect on the GS enzyme activity of roots, while leaves were more sensitive to low N condition than roots (Table 1).

**Table 1:** Effects of different N application rates on GS, GOGAT, GDH, and SPC of sugarcane at Stage 1 in 2016.

Treatments	GS activity		GOGAT activity		GDH activity		SPC	
				
	Leaf	Root	Leaf	Root	Leaf	Root	Leaf	Root
N1	39.11 ± 0.390c	10.72 ± 0.040a	71.23 ± 3.330c	61.93 ± 2.030c	158.21 ± 2.09b	77.63 ± 3.87c	0.180 ± 0.002c	0.153 ± 0.003b
N2	47.26 ± 0.120a	10.78 ± 0.050a	119.30 ± 2.180b	115.46 ± 3.010a	203.08 ± 3.73a	106.89 ± 2.04a	0.255 ± 0.002b	0.183 ± 0.003a
N3	43.17 ± 0.230b	9.81 ± 0.420b	150.35 ± 5.296a	104.60 ± 0.939b	154.41 ± 2.299b	97.81 ± 3.462b	0.270 ± 0.005a	0.139 ± 0.002c


*N1 was 225 kg/hm^*2*^ urea, N2 was 450 kg/hm^*2*^ urea, N3 was 675 kg/hm^*2*^ urea. Stage 1, the early elongation stage. GS activity (Ug^-*1*^), glutamine synthetase activity. GOGAT activity (nmolmin^-*1*^g^-*1*^FW), glutamate synthase activity. GDH activity (nmolmin^-*1*^g^-*1*^FW), glutamate dehydrogenase activity. SPC (mgg^-*1*^), soluble protein content. Different lowercase letters in the same column indicate significant differences (*P* < 0.05) between the different N application rates.*

In Figure 1, there were significant differences among the sugarcane varieties in response to N application. The SPC in the root of NCo 376 was significantly higher than that in the other varieties under N1 (Figure 1D). Compared with N2, YT94-128, and GT42 had the highest decrease in GS and GDH activities in roots among the nine varieties under N1, which decreased by 25.52% and 60.89%, respectively (Figure 1A,C). This indicates that the N assimilation capacity of YT94-128 and GT42 decreased significantly when the supply of N fertilizer was low. The GS activity of leaves and the SPC of the root in ROC22 under N2 were significantly higher than those in the other varieties (Figure 1A,D). The GS activity of leaves and SPC of roots in YT94-128 were significantly higher than those in the other varieties under N3 (Figure 1A,D). Compared with N2, LC05-136 had a largest decrease in the GS and GOGAT activities of roots under N3 among the nine varieties, which decreased by 33.49% and 44.87%, respectively (Figure 1A,B). It seemed that when LC05-136 was supplied with a large amount of N fertilizer, the activities of key enzymes involved in N metabolism decreased, which resulted in a decreased N assimilation capacity.

#### The Late Elongation Stage (Stage 2)

In Table 2, compared with N2, the GS, GOGAT, GDH activities and SPC of leaves and roots decreased significantly under low N application rate. The SPC of leaves and roots under N2 was obviously higher than that of the other treatments. Compared with N2, the activities of GS, GOGAT, GDH, and SPC under N3 were lower in the leaves. Leaves and roots under N2 had higher key enzyme activities of N metabolism and a powerful N assimilation capacity than that of N1 and N3.

**Table 2:** Effects of different N application rates on GS, GOGAT, GDH and SPC of sugarcane at Stage 2 in 2016.

Treatments	GS activity		GOGAT activity		GDH activity		SPC	
				
	Leaf	Root	Leaf	Root	Leaf	Root	Leaf	Root
N1	13.54 ± 0.060c	5.38 ± 0.070c	77.98 ± 1.000c	72.99 ± 0.850b	95.48 ± 1.220c	86.04 ± 1.050b	1.411 ± 0.007c	0.481 ± 0.004b
N2	16.06 ± 0.060a	6.49 ± 0.040a	97.77 ± 1.520a	83.30 ± 0.840a	115.11 ± 1.170a	97.51 ± 0.500a	1.492 ± 0.009a	0.524 ± 0.002a
N3	15.04 ± 0.160b	6.11 ± 0.020b	86.51 ± 0.501b	79.61 ± 0.459a	99.61 ± 1.117b	96.71 ± 1.540a	1.437 ± 0.007b	0.455 ± 0.006c


*N1 was 225 kg/hm^*2*^ urea, N2 was 450 kg/hm^*2*^ urea, N3 was 675 kg/hm^*2*^ urea. Stage 2, the late elongation stage. GS activity (Ug^-*1*^), glutamine synthetase activity. GOGAT activity (nmolmin^-*1*^g^-*1*^FW), glutamate synthase activity. GDH activity (nmolmin^-*1*^g^-*1*^FW), glutamate dehydrogenase activity. SPC (mgg^-*1*^), soluble protein content. Different lowercase letters in the same column indicate significant differences (*P* < 0.05) between the different N application rates.*

**FIGURE 2:**
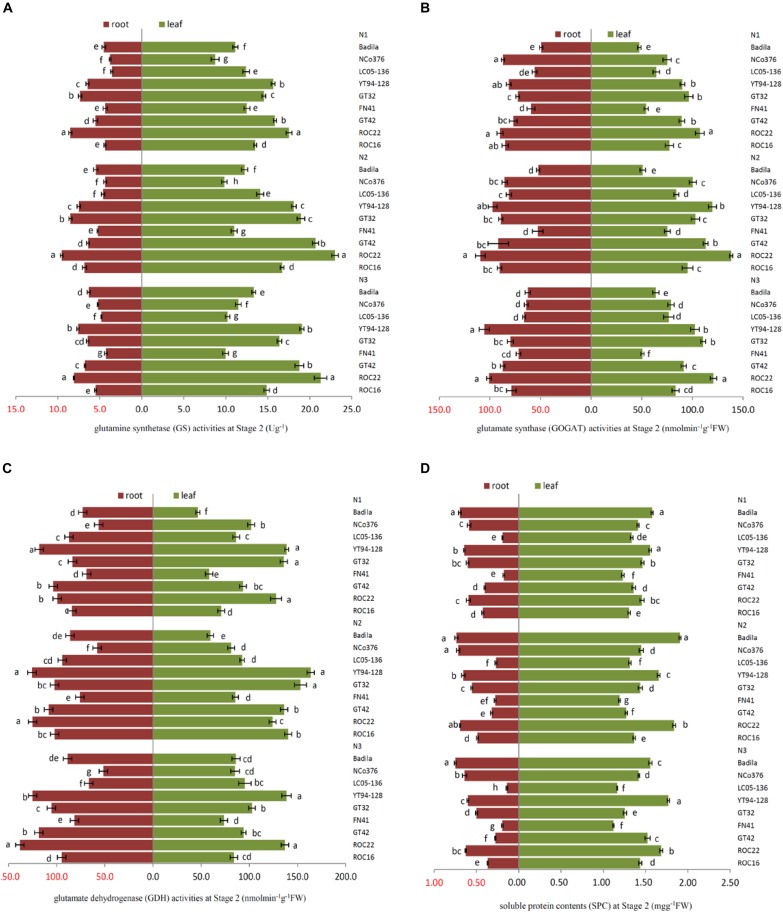
Effects of different N application rates on N assimilation enzyme activities at Stage 2 in 2016. **(A)** GS (glutamine synthetase activity), **(B)** GOGAT (glutamate synthase activity), **(C)** GDH (glutamate dehydrogenase activity), **(D)** SPC (content of soluble protein). N1 was 225 kg/hm^2^ urea, N2 was 450 kg/hm^2^ urea, N3 was 675 kg/hm^2^ urea. Stage 2, the late elongation stage. Different lowercase letters represent significant differences between the different varieties with respect to the same N application rates (*P* < 0.05).

Under N1, the SPC of leaves of FN41, ROC16 was significantly lower than that of other varieties (Figure 2D). Compared with N2, under N1, ROC16 exhibited the largest decline in the GS activity of roots, which decreased by 35.76% (Figure 2A), it suggested that the N assimilation capacity of ROC16 was low under low N. The GS and GOGAT activities of leaves of ROC22 were significantly higher than those of other varieties under N1, N2, and N3 (Figure 2A,B), suggesting a higher N assimilation ability. In Figure 2D, under N3, the SPC of leaves of YT94-128 and ROC22 was significantly higher than that of other varieties. The SPC of leaves and roots of FN41 and LC05-136 was significantly lower than that of other varieties. Compared with N2, LC05-136 and FN41 under N3 showed the largest reduction of GS and GOGAT activities in the leaf among the nine varieties, i.e., decreases by 27.53% and 32.60%, respectively (Figure 2A,B). The varieties with the largest reduction in GOGAT and GDH activities in roots under N3 were NCo376 and LC05-136, i.e., respective decreases of 25.06% and 29.21% (Figure 2B,C). These results suggest that the N assimilation capacity of LC05-136, FN41, and NCo376 decreased under higher N application.

#### Correlation of Key Enzymes Activities Between Leaf and Root in Stage 1 and Stage 2

Table 3 showed that in Stage 1, only the activity of GS in the leaf was significantly correlated with that in the root among three enzymes of GS, GOGAT and GDH, and so was SPC. While, in Stage 2, the significant correlation of activity between leaf and root was observed in each of the above three enzymes, and so was SPC.

**Table 3:** Pearson’s correlation coefficients among enzymes activities between leaves and roots at Stage1 and Stage 2 in 2016.

	Indictors		Correlation coefficients
		
	Leaf	Root	
Stage 1	LGS	RGS	0.703*
	LGOGAT	RGOGAT	0.498
	LGDH	RGDH	-0.161
	LSPC	RSPC	0.097
Stage 2	LGS	RGS	0.912**
	LGOGAT	RGOGAT	0.950**
	LGDH	RGDH	0.774*
	LSPC	RSPC	0.868**


*Stage 1, the early elongation stage. Stage 2, the late elongation stage. LGS, leaf glutamine synthetase activity; RGS, root glutamine synthetase activity; LGOGAT, leaf glutamate synthase activity; RGOGAT, root glutamate synthase activity; LGDH, leaf glutamate dehydrogenase activity; RGDH, root glutamate dehydrogenase activity; LSPC, leaf soluble protein content; RSPC, root soluble protein content. ^∗^ and ^∗∗^ indicate significant differences at the 0.05 and 0.01 levels, respectively.*

### Photosynthesis, Fluorescence Indicators and Chlorophyll Relative Content of Sugarcane Under Different N Application Rates

In Stage 1 (Table 4), the SPAD value and Fv/Fm under N1 were significantly lower than those under N2 and N3. Pn of N2 was significantly higher than that under N1 and N3. The Pn and Fv/Fm of ROC22 were significantly higher than those of other varieties under N2. The SPAD value and Pn of YT94-128 were significantly higher than those of other varieties under N3.

**Table 4:** Effects of different N application rates on SPAD, Pn, and Fv/Fm of sugarcane at Stage 1 in 2016.

	N1			N2			N3		
			
	SPAD	Pn	Fv/Fm	SPAD	Pn	Fv/Fm	SPAD	Pn	Fv/Fm
Variety/indicator
ROC16^1^	43.2 ± 0.608b	25.9 ± 0.596bc	0.780 ± 0.004c	47.9 ± 0.549ab	27.3 ± 0.321c	0.807 ± 0.004c	46.5 ± 0.578cd	22.2 ± 0.381f	0.772 ± 0.004d
ROC22^1^	47.4 ± 0.491a	28.9 ± 0.523a	0.815 ± 0.004a	49.8 ± 0.376a	32.1 ± 0.860a	0.847 ± 0.005a	48.8 ± 0.379b	27.8 ± 1.094b	0.820 ± 0.002ab
GT42^1^	47.4 ± 0.219a	27.3 ± 1.010ab	0.808 ± 0.002ab	49.4 ± 0.467a	29.4 ± 0.453b	0.831 ± 0.006b	47.9 ± 0.233bc	25.4 ± 0.942c	0.809 ± 0.005b
FN41^1^	43.1 ± 0.521b	23.3 ± 0.639de	0.743 ± 0.003de	46.1 ± 0.467b	24.6 ± 0.799d	0.756 ± 0.006e	45.4 ± 0.348d	23.3 ± 1.033cde	0.755 ± 0.004e
GT32^1^	43.7 ± 0.524b	24.8 ± 0.798cd	0.786 ± 0.006c	48.0 ± 0.606a	26.5 ± 0.527c	0.789 ± 0.003d	46.2 ± 0.467cd	24.8 ± 0.404cd	0.795 ± 0.001c
YT94-128^1^	43.2 ± 0.612b	26.6 ± 0.295bc	0.803 ± 0.001b	44.1 ± 0.926c	27.1 ± 0.446c	0.811 ± 0.004c	53.6 ± 0.436a	30.6 ± 0.818a	0.828 ± 0.009a
LC05-136^1^	42.3 ± 0.393b	21.6 ± 0.705ef	0.748 ± 0.003d	42.6 ± 0.549cd	21.5 ± 0.447e	0.753 ± 0.006e	45.5 ± 0.448d	22.8 ± 0.252de	0.749 ± 0.004ef
NCo376^1^	37.8 ± 0.696c	20.1 ± 0.599fg	0.735 ± 0.003e	41.1 ± 0.606d	20.2 ± 0.486ef	0.738 ± 0.004f	42.8 ± 1.212e	21.9 ± 0.602f	0.748 ± 0.003ef
Badila^1^	28.3 ± 0.656d	18.5 ± 0.267g	0.710 ± 0.003f	29.3 ± 0.677e	19.4 ± 0.425f	0.720 ± 0.001g	31.6 ± 1.317f	22.7 ± 0.491de	0.742 ± 0.002f
SPAD^2^		41.8 ± 1.085*b*			44.3 ± 1.187*a*			45.4 ± 1.118*a*	
Pn^2^		24.1 ± 0.668*b*			25.3 ± 0.809*a*			24.6 ± 0.578*b*	
Fv/Fm^2^		0.770 ± 0.007*b*			0.784 ± 0.008*a*			0.780 ± 0.006*a*	


*N1 was 225 kg/hm^*2*^ urea, N2 was 450 kg/hm^*2*^ urea, N3 was 675 kg/hm^*2*^ urea. Stage 1, the early elongation stage. SPAD, leaf chlorophyll relative content. Pn (μmolm^-*2*^s^-*1*^), leaf net photosynthetic rate. Fv/Fm, leaf maximum photochemical efficiency. ^*1*^Different lowercase letters in the same column indicate significant differences (*P* < 0.05) between the different varieties with respect to the same N application rates and the same indicators. ^*2*^Different lowercase letters in the same line indicate significant differences (*P* < 0.05) between the different N application rates with respect to SPAD, Pn, or Fv/Fm. All data are presented as the mean ± SE (*n* = 3).*

In Stage 2 (Table 5), the SPAD value and Fv/Fm under N3 were significantly higher than those under N2 and N1. The Fv/Fm of ROC22 was significantly higher than those of other varieties under N1. Compared with N2, the SPAD value of ROC16 exhibited the largest decline under N1 (13.90%), the Pn of YT94-128 exhibited the largest increase under N3 (54.80%).

**Table 5:** Effects of different N application rates on SPAD, Pn, and Fv/Fm of sugarcane at Stage 2 in 2016.

	N1			N2			N3		
	
	SPAD	Pn	Fv/Fm	SPAD	Pn	Fv/Fm	SPAD	Pn	Fv/Fm
Variety/indicator
ROC16^1^	37.8 ± 0.231b	20.8 ± 1.217abc	0.726 ± 0.003c	43.9 ± 0.321b	17.8 ± 0.306b	0.719 ± 0.001d	44.1 ± 0.176c	20.3 ± 0.233abc	0.692 ± 0.002f
ROC22^1^	41.1 ± 0.328a	25.7 ± 0.348a	0.760 ± 0.001a	46.6 ± 0.520a	26.3 ± 1.789a	0.759 ± 0.003b	47.5 ± 0.504a	23.4 ± 0.801a	0.754 ± 0.003bcd
GT42^1^	40.5 ± 0.470a	14.8 ± 2.403d	0.741 ± 0.003b	42.9 ± 0.240b	16.5 ± 3.136b	0.711 ± 0.002e	45.6 ± 0.404bc	14.3 ± 0.437c	0.770 ± 0.004a
FN41^1^	37.8 ± 0.578b	15.3 ± 0.769cd	0.727 ± 0.002c	39.2 ± 0.624c	17.0 ± 1.453b	0.613 ± 0.003g	42.1 ± 0.467d	16.0 ± 1.909bc	0.719 ± 0.003e
GT32^1^	41.6 ± 0.458a	25.0 ± 2.074a	0.722 ± 0.002c	43.5 ± 0.318b	25.8 ± 2.162a	0.727 ± 0.002c	42.3 ± 0.524d	24.5 ± 0.681a	0.759 ± 0.001b
YT94-128^1^	41.7 ± 0.416a	22.7 ± 3.580ab	0.706 ± 0.005d	44.0 ± 0.416b	17.7 ± 1.742b	0.761 ± 0.002b	47.1 ± 0.945ab	27.4 ± 4.107a	0.757 ± 0.002bc
LC05-136^1^	38.1 ± 0.379b	13.9 ± 1.828d	0.710 ± 0.003d	40.4 ± 0.384c	18.5 ± 0.351b	0.770 ± 0.002a	39.2 ± 0.448e	23.0 ± 1.189ab	0.725 ± 0.002e
NCo376^1^	34.7 ± 0.462c	14.4 ± 0.710d	0.638 ± 0.005e	36.3 ± 0.706d	17.4 ± 0.784b	0.703 ± 0.003f	36.3 ± 0.723f	15.9 ± 2.908bc	0.748 ± 0.004cd
Badila^1^	30.3 ± 0.285d	18.0 ± 0.285bcd	0.732 ± 0.003bc	32.3 ± 0.240e	18.6 ± 0.551b	0.767 ± 0.001a	37.6 ± 0.338ef	23.4 ± 3.514a	0.745 ± 0.005d
SPAD^2^		38.2 ± 0.706*c*			41.0 ± 0.837*b*			42.4 ± 0.768*a*
Pn^2^		17.9 ± 1.005*b*			19.5 ± 0.832*ab*			20.9 ± 1.040*a*
Fv/Fm^2^		0.718 ± 0.006*b*			0.726 ± 0.009*b*			0.741 ± 0.005*a*


*N1 was 225 kg/hm^*2*^ urea, N2 was 450 kg/hm^*2*^ urea, N3 was 675 kg/hm^*2*^ urea. Stage 2, the late elongation stage. SPAD, leaf chlorophyll relative content. Pn (μmolm^-*2*^s^-*1*^), leaf net photosynthetic rate. Fv/Fm, leaf maximum photochemical efficiency. ^*1*^Different lowercase letters in the same column indicate significant differences (*P* < 0.05) between the different varieties with respect to the same N application rates and the same indicators. ^*2*^Different lowercase letters in the same line indicate significant differences (*P* < 0.05) between the different N application rates to SPAD, Pn, or Fv/Fm. All data are presented as the mean ± SE (*n* = 3).*

As can be seen, ROC22 had high leaf chlorophyll relative content and high photosynthetic capacities under low N stress and moderate N application rate. ROC16 maintained a low leaf chlorophyll relative content and showed low tolerance to low N stress. YT94-128, which had a strong photosynthetic capacity under high N fertilizer application, was more suitable for a high N environment.

### Effects of Different N Application Rates on PNA, PDW, and NUE

#### PNA and PDW at Stage 1 and Stage 2

As shown in Table 6, at Stage 1 and Stage 2, PNA under N2 and N3 was significantly higher than that under N1. PDW under N2 was significantly higher than that under N1 and N3, indicating that sugarcane could absorb a higher percentage of N at a moderate dose of N than at the two other doses and also produced more dry matter.

**Table 6:** Effects of different N application rates and varieties on PNA and PDW of sugarcane at Stage 1 and Stage 2 in 2016.

Variety/indicator	Stage 1					
	
	N1		N2		N3	
			
	PNA	PDW	PNA	PDW	PNA	PDW
ROC16^1^	0.31 ± 0.01ef	89.54 ± 1.36e	0.44 ± 0.01d	114.57 ± 2.36e	0.43 ± 0.01d	97.27 ± 2.46d
ROC22^1^	0.66 ± 0.01a	154.88 ± 2.58a	0.69 ± 0.01b	156.67 ± 1.92c	0.67 ± 0.02b	135.99 ± 3.04b
GT42^1^	0.25 ± 0.01g	67.55 ± 1.93f	0.52 ± 0.01c	125.77 ± 3.35d	0.66 ± 0.01b	136.67 ± 2.67b
FN41^1^	0.32 ± 0.01e	94.52 ± 1.38e	0.41 ± 0.01e	114.02 ± 2.14e	0.24 ± 0.01f	59.79 ± 2.13e
GT32^1^	0.59 ± 0.01b	149.36 ± 1.79a	1.03 ± 0.01a	230.16 ± 3.27a	0.65 ± 0.01b	183.60 ± 2.64a
YT94-128^1^	0.48 ± 0.02c	134.50 ± 4.07b	0.70 ± 0.01b	169.07 ± 3.18b	0.98 ± 0.02a	192.30 ± 4.27a
LC05-136^1^	0.33 ± 0.01e	109.99 ± 3.38d	0.36 ± 0.01f	111.90 ± 2.93e	0.33 ± 0.01e	94.81 ± 2.07d
NCo376^1^	0.28 ± 0.01f	109.03 ± 3.54d	0.27 ± 0.01g	85.63 ± 1.48f	0.33 ± 0.01e	97.30 ± 3.97d
Badila^1^	0.44 ± 0.01d	125.85 ± 2.62c	0.51 ± 0.01c	123.88 ± 2.90d	0.58 ± 0.01c	126.66 ± 2.88c
PNA^2^	0.41 ± 0.03b		0.55 ± 0.04a		0.54 ± 0.04a	
PDW^2^	115.02 ± 5.38c		136.85 ± 7.96a		124.93 ± 8.03b	

**Variety/indicator**	**Stage 2**					
	
	**N1**		**N2**		**N3**	
			
	**PNA**	**PDW**	**PNA**	**PDW**	**PNA**	**PDW**

ROC16^1^	1.27 ± 0.01cd	312.93 ± 3.50b	1.37 ± 0.01d	376.98 ± 8.97cd	1.37 ± 0.01c	327.75 ± 1.73c
ROC22^1^	1.68 ± 0.04b	325.71 ± 8.04b	2.31 ± 0.01b	472.13 ± 5.17b	2.27 ± 0.01a	347.16 ± 3.74bc
GT42^1^	2.03 ± 0.12a	464.57 ± 7.97a	2.78 ± 0.07a	558.71 ± 13.18a	1.69 ± 0.04b	332.12 ± 7.28bc
FN41^1^	0.75 ± 0.06f	200.00 ± 5.58d	0.94 ± 0.07ef	215.81 ± 15.34f	1.18 ± 0.07de	284.01 ± 16.33d
GT32^1^	1.10 ± 0.05de	264.39 ± 12.51c	1.89 ± 0.12c	405.83 ± 7.92c	1.80 ± 0.11b	361.64 ± 9.92b
YT94-128^1^	1.42 ± 0.05c	325.58 ± 11.00b	1.77 ± 0.08c	363.62 ± 4.70d	2.30 ± 0.02a	488.71 ± 5.34a
LC05-136^1^	0.71 ± 0.01f	183.47 ± 3.39de	0.80 ± 0.04f	208.49 ± 7.97f	1.35 ± 0.06cd	295.35 ± 12.22d
NCo376^1^	1.05 ± 0.02e	250.57 ± 5.79c	1.44 ± 0.08d	310.71 ± 16.14e	1.04 ± 0.05e	250.54 ± 10.96e
Badila^1^	0.78 ± 0.04f	171.78 ± 8.17e	1.05 ± 0.05e	225.26 ± 5.90f	1.24 ± 0.07cd	227.89 ± 12.12e
PNA^2^	1.20 ± 0.08b		1.59 ± 0.12a		1.58 ± 0.09a	
PDW^2^	277.67 ± 17.16c		348.61 ± 22.60a		323.91 ± 14.32b	


*N1 was 225 kg/hm^*2*^ urea, N2 was 450 kg/hm^*2*^ urea, N3 was 675 kg/hm^*2*^ urea. Stage 1, the early elongation stage. Stage 2, the late elongation stage. PNA, sugarcane plant N accumulation (gplant^-*1*^). PDW, plant dry weight (g). ^*1*^Different lowercase letters in the same column indicate significant differences (*P* < 0.05) between the different varieties with respect to the same N application rates and the same indicators. ^*2*^Different lowercase letters in the same line indicate significant differences (*P* < 0.05) between the different N application rates to PNA and PDW. All data are presented as the mean ± SE (*n* = 3).*

Table 6 shows that at Stage 1, under N1, PDW and PNA of ROC22 and GT32 were significantly higher than that of other varieties. Under N2, PNA and PDW of NCo376 was significantly lower than that of other varieties. Under N3, PNA of YT94-128 was significantly higher than that of other varieties, PNA and PDW of FN41 was significantly lower than that of other varieties. At Stage 2, under N1, PNA of ROC22 and GT42 were significantly higher than those of other varieties. PNA and PDW of ROC22 and GT42 were significantly higher than those of other varieties under N2. Under N3, PDW of YT94-128 was significantly higher than those of other varieties, and PNA of YT94-128 and ROC22 was significantly higher than that in other varieties. These results suggested that ROC22 has a strong adaptability to moderate and low doses of N, absorbed more N and produced more dry matter. YT94-128 was shown to be more tolerant at a high N level.

#### PNA, PDW, and NUE at Stage 3

As we can see in Table 7, at Stage 3, PDW under N2 was significantly higher than that under N1 but was not significantly different from that under N3. PNA under N2 and N3 was significantly higher than that under N1. NUE under N3 was significantly lower than that under N2 and N3.

**Table 7:** Effects of different N application rates on PNA, PDW, and NUE of sugarcane at Stage 3 in 2016.

	N1				N2			N3			
					
	PNA	PDW	NUE	DRNUE (%)	PNA	PDW	NUE	PNA	PDW	NUE	DRNUE (%)
Variety/indicator
ROC16^1^	0.62 ± 0.01f	367.31 ± 4.43e	124.34 ± 3.79bc	5.40	1.06 ± 0.01g	528.08 ± 3.45e	131.44 ± 1.48d	1.49 ± 0.02e	588.70 ± 7.29c	104.82 ± 2.08b	20.25
ROC22^1^	1.15 ± 0.01a	606.79 ± 5.53a	163.71 ± 4.73a	10.20	1.93 ± 0.03b	840.03 ± 9.90a	182.31 ± 4.25a	2.74 ± 0.01a	835.90 ± 3.94a	120.19 ± 1.13a	34.07
GT42^1^	0.72 ± 0.01de	479.57 ± 5.45c	106.86 ± 4.66cd	35.07	1.55 ± 0.01c	738.01 ± 1.75b	164.58 ± 0.75b	2.18 ± 0.07b	702.06 ± 21.08b	99.29 ± 6.02b	39.67
FN41^1^	0.72 ± 0.03de	394.94 ± 15.61d	100.14 ± 13.34de	26.61	1.49 ± 0.03d	595.70 ± 10.55d	136.45 ± 4.53cd	1.47 ± 0.05e	505.72 ± 17.08d	65.13 ± 4.88c	52.27
GT32^1^	0.82 ± 0.01c	505.22 ± 2.90b	128.13 ± 2.48b	9.90	1.39 ± 0.01e	686.67 ± 7.38c	142.21 ± 3.17c	2.00 ± 0.03c	710.99 ± 12.22b	101.62 ± 3.49b	28.54
YT94-128^1^	0.88 ± 0.01b	471.23 ± 4.36c	99.90 ± 3.73de	42.07	2.11 ± 0.01a	756.18 ± 6.12b	172.46 ± 2.63b	1.78 ± 0.02d	803.10 ± 7.33a	128.21 ± 2.09a	25.66
LC05-136^1^	0.68 ± 0.02e	368.06 ± 9.22e	122.38 ± 7.88bc	-23.90	0.86 ± 0.01h	455.01 ± 2.97f	98.77 ± 1.27e	1.16 ± 0.02f	485.25 ± 7.14d	74.39 ± 2.04c	24.68
NCo376^1^	0.76 ± 0.01d	391.96 ± 6.78d	99.05 ± 5.80de	-27.26	1.17 ± 0.02f	457.41 ± 6.20f	77.83 ± 2.66f	1.39 ± 0.02e	441.06 ± 7.15e	47.14 ± 2.04d	39.43
Badila^1^	0.87 ± 0.02b	305.95 ± 6.13f	83.11 ± 5.24e	16.49	1.37 ± 0.02e	440.60 ± 7.60f	99.52 ± 3.26e	1.72 ± 0.03d	470.80 ± 9.18de	74.88 ± 2.62c	24.76
PNA^2^	0.80 ± 0.03c		1.44 ± 0.07b	1.77 ± 0.09a	
PDW^2^	432.34 ± 17.03b		610.85 ± 27.76a	615.95 ± 28.03a	
NUE^2^	114.18 ± 4.73b		133.95 ± 6.72a	90.63 ± 5.03c	


*N1 was 225 kg/hm^*2*^ urea, N2 was 450 kg/hm^*2*^ urea, N3 was 675 kg/hm^*2*^ urea. Stage 3, the technical maturation stage. PNA (gplant^-*1*^), sugarcane plant N accumulation. PDW (g), plant dry weight. NUE (gDWg^-*1*^N), nitrogen use efficiency. DRNUE, Decline ratio of NUE. ^*1*^Different lowercase letters in the same column indicate significant differences between the different varieties with respect to the same N application rates and the same indicators (*P* < 0.05). ^*2*^Different lowercase letters in the same line indicate significant differences between different N application rates to PNA, PDW, and NUE (*P* < 0.05). All data are presented as the mean ± SE (*n* = 6).*

As shown in Table 7, the values of PNA, PDW, and NUE of NCo376, LC05-136, FN41, and Badila were relatively low under different N application rates than that of other varieties under N3. Compared with N2, the largest decrease of PNA, PDW, and NUE under N1 was observed in YT94-128, with respective decreases of 58.29%, 37.68%, and 42.07%. Under N1 and N2, PDW, and NUE of ROC22 were significantly higher than those of other varieties. PNA of ROC22 was significantly higher than that of other varieties under N3. Compared with N2, the largest decrease of PDW and NUE under N3 was observed in FN41, i.e., respective decreases of 15.10% and 52.27%.

The above results suggested that ROC22 has a strong adaptability to different N application rates. Besides, PDW and NUE of ROC22 were relatively high regardless of low or high N application rates. In addition, ROC22 was more tolerant to low N stress, and thus the cultivation of ROC22 only requires the application of low to moderate doses of N. However, Badila was distinctive, its NUE and PDW were similar to those of NCo376, LC05-136 and FN41, while the values were quite low. Similar to ROC22, compared with N2, NUE of GT32 and ROC16 was decreased by 9.90% and 5.40% under N1 treatment, and decreased by 28.54% and 20.25% under N3 treatment, respectively (Table 7). GT32 and ROC16 did not decrease much in NUE under N1 and N3 treatments. GT32 and ROC16 were insensitive to fertilizer application. Compared with other seven varieties except ROC22, GT32 had relatively better growth based on NUE and PDW under N1 treatment. Compared with N2, NUE of GT42 and YT94-128 was decreased by 39.67% and 25.66% under N3 treatment, respectively. Compared with other N treatments, GT42 was more suitable for moderate N environment (450 kg/hm^2^ urea) based on NUE and PDW under N2 treatment. Compared with N1 treatment, YT94-128 was more suitable for moderate to high N environments (450–675 kg/hm^2^ urea) based on NUE and PDW under N2 and N3 treatments (Table 7).

### Correlations Between NUE at Stage 3 and the Other Indicators at Stage 1 and Stage 2

It is shown in Table 8 that a significant correlation was obtained between PNA at Stage 1 and NUE at Stage 3, but a non-significant correlation was observed for PDW at Stage 1 and NUE at Stage 3 (*P* < 0.05). A significant correlation was also observed between PNA at Stage 2 and NUE at Stage 3 (*P* < 0.01), and the correlation between PDW at Stage 2 and NUE at Stage 3 was significant at *P* < 0.05. These results suggested that PNA and PDW at Stage 2 could better reflect NUE at Stage 3 than the PNA and PDW at Stage 1.

**Table 8:** Pearson’s correlation coefficients among indicators of nine sugarcane varieties cultivated in a pot experiment under different N application rates at different stages in 2016.

Indicator	Pearson’s correlation coefficient
	
	NUE at Stage 3
PNA at Stage 1	0.716*
PDW at Stage 1	0.572
PNA at Stage 2	0.811**
PDW at Stage 2	0.772*


*Stage 1, the early elongation stage. Stage 2, the late elongation stage. Stage 3, the technical maturation stage. PNA, sugarcane plant N accumulation; PDW, plant dry weight; NUE, nitrogen use efficiency. ^∗^ and ^∗∗^ indicate significant differences at the 0.05 and 0.01 levels, respectively.*

### Principal Component Analysis of Physiological and Agronomic Indicators at Stage 2

**FIGURE 3:**
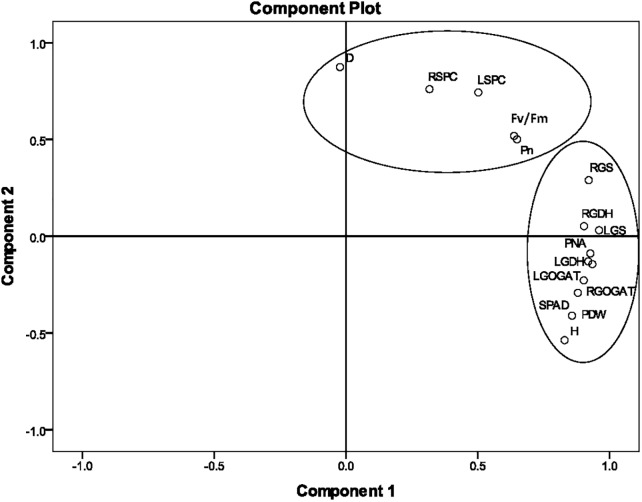
The principal component analysis (PCA) of 15 agronomic and physiological indicators at Stage 2 in 2016. The PCA shows the biplot of the first two principal components. The eigenvectors were as follows. H, plant height; D, stalk diameter; SPAD, leaf chlorophyll relative content; LGS, leaf glutamine synthetase activity; LGDH, leaf glutamate dehydrogenase activity; LGOGAT, leaf glutamate synthase activity; LSPC, content of soluble protein in the leaf; RGS, root glutamine synthetase activity; RGDH, root glutamate dehydrogenase activity; RGOGAT, root glutamate synthase activity; RSPC, content of soluble protein in the root; Pn, leaf net photosynthetic rate; Fv/Fm, leaf maximum photochemical efficiency; PNA, plant nitrogen accumulation; PDW, plant dry weight. Stage 2, the late elongation stage.

According to the correlation analysis, the PCA of agronomic and physiological indicators was concentrated on Stage 2. The loading plots of principle components 1 and 2 of the PCA analysis on 15 selected indicators obtained from the average value of nine sugarcane varieties at Stage 2 under different N application rates are illustrated in Figure 3. The 15 selected indicators were H, D, SPAD, Pn, Fv/Fm, LGS, LGDH, LGOGAT, LSPC, RGS, RGDH, RGOGAT, RSPC, PNA, and PDW. The total variance contributions for the first and second principal components were 62.37% and 20.94%, respectively, and the accumulated contribution reached 83.31%, which basically represented the original information. In the first principal component, the absolute values of the eigenvectors of H, SPAD, LGS, LGOGAT, LGDH, RGS, RGOGAT, RGDH, PNA, and PDW were relatively large, indicating that key enzyme activities of N metabolism were closely related to PDW and PNA at Stage 2. In the second principal component, the absolute values of indicators such as D, LSPC, RSPC, Pn, and Fv/Fm were relatively large, indicating that the second component mainly characterized the soluble protein content of the leaf and root and photosynthetic fluorescence indicators.

### Correlations Between PDW and NUE of Stage 3 and All the Other Indicators at Stage 2

Table 9 indicated that, PNA and PDW were significantly correlated with H, SPAD, LGS, LGOGAT, LGDH, RGS, RGOGAT, and RGDH at Stage 2. In addition, a similar significant correlation between PDW and NUE at Stage 3 and H, SPAD, LGS, LGOGAT, LGDH, RGS, RGOGAT, RGDH, PNA, and PDW at Stage 2 was observed, respectively (Table 10).

**Table 9:** Pearson’s correlation coefficients among indicators at Stage 2 in 2016.

Indictors	PNA	PDW
H	0.819**	0.904**
D	-0.048	-0.202
SPAD	0.771*	0.819**
LGS	0.929**	0.866**
LGDH	0.772*	0.777*
LGOGAT	0.865**	0.828**
LSPC	0.429	0.251
RGS	0.819**	0.696*
RGDH	0.827**	0.779*
RGOGAT	0.837**	0.850**
RSPC	0.248	0.104
Pn	0.372	0.254
Fv/Fm	0.572	0.400


*Stage 2, the late elongation stage. H, plant height; D, stalk diameter; SPAD, leaf chlorophyll relative content; LGS, leaf glutamine synthetase activity; LGDH, leaf glutamate dehydrogenase activity; LGOGAT, leaf glutamate synthase activity; LSPC, leaf soluble protein content; RGS, root glutamine synthetase activity; RGDH, root glutamate dehydrogenase activity; RGOGAT, root glutamate synthase activity; RSPC, root soluble protein content; Pn, leaf net photosynthetic rate; Fv/Fm, leaf maximum photochemical efficiency; PNA, plant nitrogen accumulation; PDW, plant dry weight. ^∗^ and ^∗∗^ indicate significant differences at the 0.05 and 0.01 levels, respectively.*

**Table 10:** Pearson’s correlation coefficients among indicators at Stage 2 and Stage 3 in 2016.

Indicator at Stage 2	Stage 3	
	
	PDW	NUE
H	0.812**	0.747*
D	-0.045	0.012
SPAD	0.883**	0.929**
LGS	0.922**	0.947**
LGDH	0.864**	0.798*
LGOGAT	0.903**	0.815**
LSPC	0.368	0.325
RGS	0.923**	0.884**
RGDH	0.845**	0.924**
RGOGAT	0.841**	0.791*
RSPC	0.215	0.073
Pn	0.613	0.652
Fv/Fm	0.499	0.558
PNA	0.897**	0.811**
PDW	0.827**	0.772*


*Stage 2, the late elongation stage. Stage 3, the technical maturation stage. H, plant height; D, stalk diameter; SPAD, leaf chlorophyll relative content; LGS, leaf glutamine synthetase activity; LGDH, leaf glutamate dehydrogenase activity; LGOGAT, leaf glutamate synthase activity; LSPC, leaf soluble protein content; RGS, root glutamine synthetase activity; RGDH, root glutamate dehydrogenase activity; RGOGAT, root glutamate synthase activity; RSPC, root soluble protein content; Pn, leaf net photosynthetic rate; Fv/Fm, leaf maximum photochemical efficiency; PNA, plant nitrogen accumulation; PDW, plant dry weight. ^∗^ and ^∗∗^ indicate significant differences at the 0.05 and 0.01 levels, respectively.*

### Screening Characteristic Indictors for Estimation of Sugarcane NUE

In order to establish a comprehensive evaluation method of sugarcane NUE, stepwise regression analysis was carried out, and NUE was predicted based on indicators (H, D, SPAD, LGS, LGOGAT, LGDH, LSPC, RGS, RGOGAT, RGDH, RSPC, Pn, Fv/Fm, PNA, PDW) at Stage 2. The stepwise regression equation was as follows: Y = -80.50 + 5.52X_1_ + 3.64X_2_ - 0.12X_3_ (*R*^2^ = 0.99, *P* < 0.01), indicating that the model simulation results were trustworthy. In the equation, Y is the predicted value of NUE at Stage 3, and X_1_, X_2_, and X_3_ are LGS, SPAD, and PDW, respectively, at Stage 2. The three variables (LGS, SPAD, and PDW) at Stage 2 could determine 99% of the total variation of the F value (138.53), and the overall model was significant. This suggested that sugarcane NUE could be estimated based on leaf GS activity, leaf SPAD and PDW at Stage 2.

**FIGURE 4:**
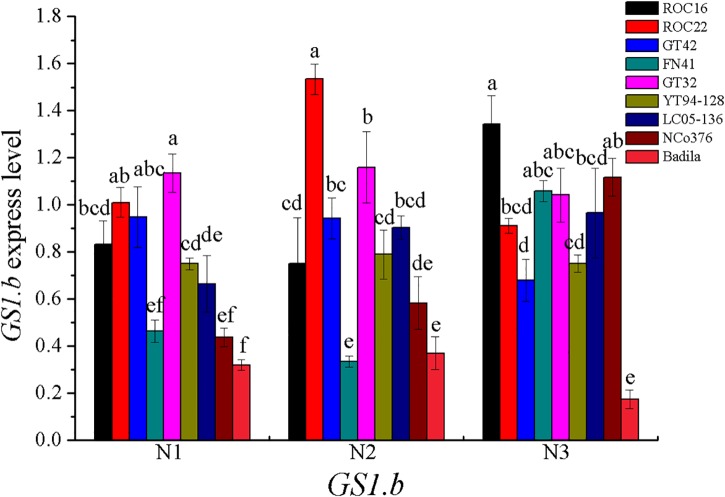
*GS1.b* expression under different N application rates at Stage 2 in 2016. N1 was 225 kg/hm^2^ urea, N2 was 450 kg/hm^2^ urea, N3 was 675 kg/hm^2^ urea. Stage 2, the late elongation stage. Different lowercase letters represent significant differences between the different varieties with respect to the same N application rates (*P* < 0.05).

In most of higher plants, *GS* can be divided into two main isoforms, *GS1* (cytoplasmic type) and *GS2* (plastid type) (Morey et al., 2002). *GS1* are encoded by a gene family (*GS1.a*, *GS1.b*, *GS1.c*) (Nogueira et al., 2005), and *GS2* are encoded by a single gene (Lam et al., 1996; Nogueira et al., 2005). In addition, Eduardo found that gene for dodecameric *GS* (*GSI*) were observed in sugarcane (Nogueira et al., 2005). In our research, the correlation between *GS1.b* expression and LGS activity was significant (Table 11). The *GS1.b* expression in various sugarcane varieties under different N concentration treatments was shown in the Figure 4.

**Table 11:** Pearson’s correlation coefficients between LGS and the expressions of *GS* family genes at Stage 2 in 2016.

Genes	Pearson’s correlation coefficient
	
	LGS
*GS1.a*	0.272
*GS1.b*	0.487*
*GS1.c*	-0.231
*GS2*	0.228
*GSI*	0.380


*Stage 2, the late elongation stage. *GS* family genes: *GS1* (*GS1.a*, *GS1.b*, *GS1.c*), *GS2*, and *GSI*. LGS, leaf glutamine synthetase activity. ^∗^ indicate significant differences at the 0.05 levels.*

Path analysis was conducted to further analyze the direct and indirect effect of each indictor based on the results of stepwise regression analysis. As shown in Table 12, among the three indicators (LGS, SPAD, and PDW) at Stage 2, LGS had the most direct effect on NUE at Stage 3, followed by SPAD and PDW. Indirect path coefficient analysis showed that both SPAD and PDW had a positive effect on NUE through LGS. The above results suggested that LGS is the most critical indicator for prediction of sugarcane NUE.

**Table 12:** Path analysis among indicators (LGS, SPAD, and PDW) at Stage 2 in 2016.

Independent variable	Total effect coefficient	Direct effect coefficient	
			Indirect effect coefficient
			
			X_1_	X_2_	X_3_
X_1_	0.947	0.802	–	0.480	-0.335
X_2_	0.929	0.567	0.680	–	-0.317
X_3_	0.772	-0.387	0.695	0.464	–


*Stage 2, the late elongation stage. X_*1*_, LGS (leaf glutamine synthetase activity); X_*2*_, SPAD (leaf chlorophyll relative content); X_*3*_, PDW (plant dry weight).*

**FIGURE 5:**
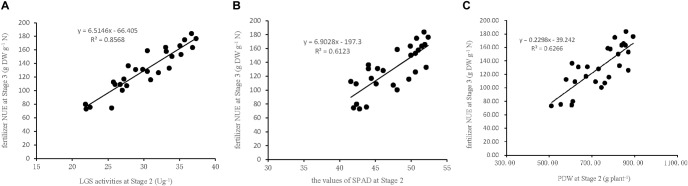
Relationships between NUE at Stage3 and the indicators of LGS, SPAD, and PDW at Stage 2 in the field experiment in 2017. **(A)** LGS (leaf glutamine synthetase activity), **(B)** SPAD (leaf chlorophyll relative content), **(C)** PDW (plant dry weight). Stage 2, the late elongation stage. Stage 3, the technical maturation stage.

To verify the accuracy of the indicators used in the model to estimate NUE, three linear regression equations were established based on the indicators LGS, SPAD, and PDW collected at Stage 2 in the field experiment in 2017. The data were used to establish the (X) of Stage 2 and (Y) of Stage 3 (Figure 5): Y (NUE) = 6.51X (LGS)-66.41 (*R*^2^ = 0.86, *P* < 0.01), Y (NUE) = 6.90X (SPAD)-197.3 (*R*^2^ = 0.61, *P* < 0.01), and Y (NUE) = 0.23X (PDW)-39.24 (*R*^2^ = 0.63, *P* < 0.01). This indicates that each of the three indicators used in the linear regression equation is suitable for the estimation of sugarcane NUE, however the LGS was the most critical indicator because of its stronger relationship with NUE than that of SPAD and PDW. This suggested that the characteristic indicators, selected based on the pot experiment for the estimation of sugarcane NUE, are consistent with the field experiment.

## Discussion

### The Physiological and Agronomic Changes in Sugarcane Responsive to the Different N Application Rate

N is closely related to plant growth and development and affects cell regulation and metabolism. The supply of N determines the crop yield (Xu et al., 2012). Usually, nitrate-N and ammonium-N absorbed by plants are transformed into amino acid by enzymes of N assimilation and then to proteins that can be utilized by plants. Therefore, the enzyme activity of N assimilation would have a great effect on the N metabolic rate, leading to differences in NUE (Jawad et al., 2017). The activities of N assimilation key enzymes GS, GOGAT, and GDH in N metabolism reflect the strength of N assimilation of crops. Different N application rates affect the activities of the key N assimilation enzymes in sugarcane (Jawad et al., 2017). In our experiment, N assimilation enzymes in the leaf and root at the late elongation stage exhibited higher activity under the moderate N application rate, along with higher SPC, SPAD, Pn, and Fv/Fm resulting in higher PDW and NUE at the technical maturation stage. In addition, the activities of GS, GOGAT, and GDH of the high NUE varieties were generally higher than those with low NUE. These findings are similar to the previous report. In barley, activities of GS, GOGAT, and GDH in the root or leaf of plantlets increased with increasing N-content (0–2 mmol L^-1^) under hydroponic culture, and varieties with high NUE had higher GS, GOGAT, and GDH activities (Jawad et al., 2017). Fluorescence indicators, which are dramatically affected under biotic or abiotic stresses, are usually regarded as indicators of stress tolerance (Chai et al., 2016). It was reported that the chlorophyll content (Kumara and Bandara, 2001), photosynthesis (Meinzer and Zhu, 1998; Kumara and Bandara, 2001), maximal photochemical efficiency (Meinzer and Zhu, 1998), N content and biomass (Kumara and Bandara, 2001) decreased significantly in sugarcane under a low N supply. Our results showed that several key enzyme activities of N-metabolism, such as GS, GDH and GOGAT, were significantly decreased at early and late elongation stages under low N stress compared with those of the moderate N application rate, similar to Pn and Fv/Fm. This resulted in the obviously lower PNA and PDW but higher NUE under low N stress compared to the moderate and high doses of N. These observations might suggest that low N restricted N-assimilation, leading to photo inhibition with lower potential photochemistry, and then decreased photosynthesis and Pn, accompanied by reduced N accumulation at the technical maturation stage, and the decreased photosynthesis eventually resulted in lower biomass. In addition, the decreased amount of N that accumulated in the plant was greater than that of biomass after low N stress, resulting in higher NUE.

The final product of N-metabolism is protein, while the content of soluble protein can reflect the process of N-metabolism to a certain extent, and then the senescence state of the leaf (Weng et al., 2014). Our research showed, compared with the moderate and low application rates, the higher SPC of leaves under the high N application rate at Stage 1 indicated that more proteins were synthesized in the sugarcane leaf to alleviate the damage caused by high N stress. At the technical maturation stage, maximum PNA was observed in the high N application rate while the PDW of the moderate N application rate had no difference with that of high application rate, which demonstrated that a high supply of N could promote N accumulation in sugarcane but failed to further increase the biomass.

Under N stress, above-ground partitioning in crops is more sensitive to N than that of roots (Chen et al., 2016). The enzyme activities of GS and GOGAT and the SPC content of sugarcane leaves under N stress at the early/late elongation stages were higher than those of the roots in our experiment. However, N stress had a smaller effect on root GS activity and a greater effect on leaf GS and GOGAT activities. This may due to the reason that leaf is the most important organ for photosynthesis, in which N-metabolism mainly occurs, and Glutamine produced by the GS/GOGAT pathway is transferred from the leaf to the root, which inhibits the synthesis of key N-metabolism enzymes in the root. Therefore, the GS/GOGAT pathway in the leaf was more vigorous than that in the root under N stress.

### Fertilization Guidelines for Sugarcane Varieties

Applying N fertilizer in the key fertilizer requirement period, increasing the number of fertilization and enhancing efficiency fertilizers can better match N supply to crop demand and have an impact on crop NUE. In addition, genetic improvement, trait physiology, transgenic approaches, and remote sensing technologies can improve sugarcane NUE (Bell et al., 2014). However, screening crop varieties with low N tolerance and high NUE from those widely planted at present is an effective means to promote agricultural production at a lower cost of N-fertilizer input (Zhang C. et al., 2017). In the present study, the NUE differences among different varieties supplied with different dosage of N-fertilizer was analyzed to identify the NUE types of varieties under LN, NN, and HN environments. ROC22 was identified to have high NUE under low N (225 kg/hm^2^ urea). The activities of key enzymes involved in the N-metabolism of ROC22 decreased to a lesser extent under a low supply of N-fertilizer in this study, along with a high chlorophyll content in the leaf maintained photosynthesis, and relatively high Pn, Fv/Fm and soluble protein aided in the adaption to low N stress to decrease leaf senescence. These maybe are part of the important factors that its agronomic performance especially the stability of cane yield in different years and varied ecological regions is outstanding, and resulting in the largest planting in recent 15 years and at present China. Similar results of tolerance to low dosage N were obtained by Li (2011) in sand culture of ROC22. Our experiment found that the variety GT32 had a good tolerance to low N (225 kg/hm^2^ urea), GT42 was more suitable for moderate N environment (450 kg/hm^2^ urea), and YT94-128 was more suitable for middle N and high N supply (450–675 kg/hm^2^ urea) based on the analysis of key enzyme activities of N-metabolism, photosynthesis fluorescent, NUE and PDW at different growth stages. In addition, the variety of Badila had lowest PDW and NUE among nine varieties under low N supply (225 kg/hm^2^ urea). NCo376 has low PDW and NUE under low N condition (225 kg/hm^2^ urea), illustrating a low NUE variety, which consisted with Hajari et al. (2014) research. It is anticipated that these results can guide the use of fertilizer in sugarcane cultivation to promote production according to the NUE of the variety and the soil N content.

One of the purposes in this study is to reduce the applied amount of N fertilizer while maintaining or reducing the yield loss, which should result in a better input–output ratio. Indeed, an increment of the application amount of N fertilizer can increase the yield, while too much N fertilizer applied will definitely increase the input and result in the decrement of the input–output ratio. Previously, excessive application of N fertilizer in China has caused acidic soil, eutrophic water, and non-point pollution. Thus, in order to reduce the risk of environmental pollution, one of the incentive policies issued by government is to subsidize farmers if one reduces the application amount of N fertilizer during cultivation. In order to obtain subsidies and good input–output ratio, farmers may reduce the use of N fertilizer.

### Screening of NUE Characteristic Indicators for Sugarcane

N is a key limited factor for continuous sugarcane output, while excessive application of N will lead to many problems, such as soil acidification, eutrophication of water bodies and high cost. NUE is an important indicator of N uptake and utilization, and thus it attracts a lot of research (Foulkes et al., 2009; Oliveira et al., 2013; Jawad et al., 2017; Rajesh et al., 2017; Zhang C. et al., 2017). In these previous studies, the conventional calculation method of NUE (the total productivity or PDW divided by the fertilizer N applied) is biomass/N supplied (NUE = NUpE × NUtE) (Snyman et al., 2015), which took both N uptake efficiency (NUpE; plant N content/N supplied) and N utilization efficiency (NUtE; biomass/plant N content) into account. However, an increase in either soil or applied N could be equally important in determining NUE (Bell et al., 2014). Without understanding the effects of basic soil fertility on plants and the incremental responses of fertilizer N application, fertilizer NUE could not be reasonably assessed. The above calculation method does not take account of the background effect of N application rate of 0 kg/hm^2^, and may be not so suitable. To eliminate objective errors as far as possible, the calculation method of NUE adopted in this paper is that the difference between PDW with N treatment and PDW without N treatment is divided by N supply (Zhao et al., 2014).

With respect to the screening methods, Pearson’s correlation and multiple regression were used to obtain the predictive models (Pavuluri et al., 2015). Based on correlation analysis, a stepwise regression equation was established to screen the indicators and to quantify the relationship between dependent and independent variables. In Tartary buckwheat, based on correlation analysis, an optimal regression equation was built to identify eight physiological and morphological indicators (plant height, stem diameter, leaf area, root-shoot ratio, chlorophyll content, Fm, SOD activity, and NUE) (Zhang C. et al., 2017), which were also used as the screening indicators for winter wheat varieties with low N tolerance (Zhang Z.Y. et al., 2017). In rice, a combined method of stepwise regression equation and correlation analysis was used to identify five morphological and yield indicators (plant height, spikelets per panicle, seed set, 1000-grain weight, and yield per plant) to evaluate low N tolerance (Hu et al., 2015). However, in sugarcane, a stepwise regression equation was only used in the estimation of cane yield (Luo et al., 2014). In our research, principle componential analysis, correlation analysis, stepwise regression analysis and path analysis were all carried out to evaluate the physiological and agronomic parameters for the selection of characteristic indictors to predict the NUE of sugarcane.

Physiologically and genetically, NUE is a complex trait, and so far there is not an indirect selection trait in crops, which can be used for variety improvement. A better understanding of N physiology in the local crop germplasm is fundamental for genetic improvement of NUE (Bell et al., 2014). In our research, we carried out a pot- and field- experiments under low N, moderate N and high N to investigate 15 parameters of aboveground and belowground attributes, including 12 physiological parameters (SPAD, Pn, Fv/Fm, LGS, LGDH, LGOGAT, LSPC, RGS, RGDH, RGOGAT, RSPC, PNA) and three agronomic indicators (H, D, PDW), and found out LGS, SPAD, and PDW at the late elongation stage were suitable indicators for the evaluation of sugarcane NUE according to a stepwise regression equation and a linear regression equation. However, LGS was the most critical indicator, which was supported by path analysis. This is consistent with the view that GS is considered as the key regulator of NUE for maize (Oliveira et al., 2013), rice (Rajesh et al., 2017), and wheat (Zhang Z.Y. et al., 2017). However, Robinson et al. (2007) found no obvious correlation of LGS with NUE in sugarcane plantlets cultured under various N-content solutions, which may be related to the opinion that most N accumulation occurs within the 3–6 month growing period in sugarcane (Wood et al., 1996), and a later measurement in plant other than in plantlet may result in better reflection of N uptake in sugarcane. This phenomenon, i.e., weak N uptake of sugarcane in the first 3 months of the crop season, was also observed in a field experiment (Robinson et al., 2011). In addition, SPAD at the heading stage was suggested to be a good indicator for the estimation of wheat NUE by simple linear regression (Nguyen et al., 2016). Silva et al. (2012) found that SPAD values were not affected by N topdressing based on polynomial regression and simple linear correlation analyses, and there was no significant correlation between SPAD value and leaf N during the full flowering stage of Crambe. Our research found that SPAD at the late elongation stage may be another suitable indicator to predict NUE. Besides, Li (2011) thought that above-ground biomass and photosynthesis could be used as screening indicators for NUE under low N stress in sugarcane plantlets. However a weak correlation between photosynthesis at the late elongation stage and NUE was found in our research, and only plant biomass was significantly correlated with NUE.

*GS* is divided into two types, including dodecameric and octameric subunits. In our research, the dodecameric *GS* gene, *GSI*, was expressed in the leaf of sugarcane, which was similar to the previous report (Nogueira et al., 2005). We found that the correlation between *GSI* expression and LGS enzyme activity was not significant. Octameric *GS* is the best characterized type of *GS* in plants, which can be divided into *GS1* and *GS2* (Nogueira et al., 2005). *GS1* exists in roots, stems, nodules and other plant tissues, mainly assimilating primary ammonium into glutamine for transportation and re-assimilating N released by N circulation pathway; *GS2* exists in mesophyll cells and can assimilate ammonia released nitrate reduction and photorespiration (Wang et al., 2013). In many C3 plants, *GS1* is generally less abundant in photosynthetic tissues, however in the leaves of C4 plants, the relatively high abundance of *GS1* was enriched, which was assumed to play a key role in N metabolism (Nogueira et al., 2005). The previous researches reported that in the leaves, the GS1 activity in C4 crops sorghum represented about 70% of the total GS activity (Hirel and Gadal, 1982; Mcnally et al., 1983). In sugarcane, *GS1* can be important for N primary assimilation and N re-assimilation released by protein degradation in senescing leaves (Nogueira et al., 2005; James et al., 2018). In the present study, the gene expression of *GS1.b* was significantly correlated with LGS enzyme activity, thus *GS1.b* can be a potential candidate marker gene for screening NUE at Stage 2 in sugarcane.

In maize, QTLs for various agronomic traits, dependent on N availability of soil, coincide with cytosolic *GS* locus on chromosome 5 (Gallais and Hirel, 2004). If we can sequence the genes encoding the presented enzymes of N metabolism and find those SNPs that are associated with the NUE, then it should greatly increase the efficiency of NUE screening in sugarcane. However, sugarcane is an 8–10 ploid (It is not clear now) crop with multiple alleles and multiple copies of genes, and the genome of modern sugarcane cultivar has not yet been deciphered. Obvious differentially expression of gene at the RNA level may not always be corresponded to sequence difference at the DNA level. What is more, N-efficient genes are quantitative traits controlled by multiple genes. At present, the key genes for N-efficient utilization that we selected for analysis are some among them, and the remaining N-efficient genes need to be further explored and studied.

## Conclusion

In sum, the physiological data at Stage 2 were more suitable for the evaluation of sugarcane NUE than those at Stage 1. Among 15 indictors (H, D, SPAD, Pn, Fv/Fm, LGS, RGS, LGDH, RGDH, LGOGAT, RGOGAT, LSPC, RSPC, PDW, and NUE at Stage 2), LGS, SPAD and PDW were selected to predict NUE by PCA, correction analysis and stepwise regression equation. Based on three parameters, LGS, SPAD or PDW, a linear regression equation was built for the estimation of NUE in a field experiment. LGS was identified as the most critical indicator, and *GS1.b* expression was significantly correlated with LGS activity at Stage 2 in sugarcane. NUE in the low-N application rate was significantly higher than that at high-N (*P* < 0.05). The dominant variety ROC22 having been used in Chinese industry for more than 15 years and at present China, with excellent low-N tolerance (225 kg/hm^2^ urea), has high NUE. The varietiesYT94-128 and GT42 exhibited weak tolerance to low N, GT42 was more suitable for moderate N environment (450 kg/hm^2^ urea) and YT94-128 was identified to be more suitable for middle N and high N supply (450–675 kg/hm^2^ urea), while GT32 was good performing genotype for PDW and NUE under low N supply (225 kg/hm^2^ urea).

## Author Contributions

YY, LX, and YQ conceived, designed, initiated the project, and revised and approved the final version of the manuscript. YY, SG, YJ, ZL, JL, ML, JG, and YS performed the experiments and contributed to data analysis and validation. YY wrote the manuscript.

## Conflict of Interest Statement

The authors declare that the research was conducted in the absence of any commercial or financial relationships that could be construed as a potential conflict of interest.

## Footnotes

1 https://www.ncbi.nlm.nih.gov/tools/primer-blast/
